# Evolving Role of Endoscopic Retrograde Cholangiopancreatography in Management of Extrahepatic Hepatic Ductal Injuries due to Blunt Trauma: Diagnostic and Treatment Algorithms

**DOI:** 10.1155/2008/259141

**Published:** 2007-11-18

**Authors:** Nikhil P. Jaik, Brian A. Hoey, S. Peter Stawicki

**Affiliations:** ^1^Department of Surgery, St Luke's Hospital and Health Network, Bethlehem, PA 18015, USA; ^2^Regional Level I Resource Trauma Center, St Luke's Hospital and Health Network, Bethlehem, PA 18015, USA; ^3^University of Pennsylvania Trauma Network, Philadelphia, PA 19104, USA; ^4^STAR/OPUS12 Foundation, 304 Monroe Boulevard, King of Prussia, PA 19406, USA

## Abstract

Extrahepatic hepatic ductal injuries (EHDIs) due to blunt abdominal trauma are rare. Given the rarity of these injuries and the insidious onset of symptoms, EHDI are commonly missed during the initial trauma evaluation, making their diagnosis difficult and frequently delayed. Diagnostic modalities useful in the setting of EHDI include computed tomography (CT), abdominal ultrasonography (AUS), nuclear imaging (HIDA scan), and cholangiography. Traditional options in management of EHDI include primary ductal repair with or without a T-tube, biliary-enteric anastomosis, ductal ligation, stenting, and drainage. Simple drainage and biliary decompression is often the most appropriate treatment in unstable patients. More recently, endoscopic retrograde cholangiopancreatography (ERCP) allowed for diagnosis and potential treatment of these injuries via stenting and/or papillotomy. Our review of 53 cases of EHDI reported in the English-language literature has focused on the evolving role of ERCP in diagnosis and treatment of these injuries. Diagnostic and treatment algorithms incorporating ERCP have been designed to help systematize and simplify the management of EHDI. An illustrative case is reported of blunt traumatic injury involving both the extrahepatic portion of the left hepatic duct and its confluence with the right hepatic duct. This injury was successfully diagnosed and treated using ERCP.

## 1. INTRODUCTION

Injuries to the extrahepatic biliary system in blunt abdominal trauma are uncommon [1–7]. Extrahepatic hepatic ductal injuries (EHDIs) occur even less frequently [1–8]. Because of their rarity and the frequently insidious onset of symptoms, EHDIs are commonly missed during the initial trauma evaluation, and debate continues regarding the best way to diagnose and treat them [2, 9]. Diagnostic tools useful in EHDIs include computed tomography (CT), abdominal ultrasound (AUS), nuclear imaging (HIDA), percutaneous transhepatic cholangiography (PTC), and endoscopic retrograde cholangiopancreatography (ERCP).

Traditional management options in EHDI include primary repair with or without a T-tube, biliary-enteric anastomosis, ductal ligation, stenting, and drainage. Simple drainage and biliary decompression is often the most appropriate treatment option in unstable patients [2, 3]. More recently, ERCP has allowed trauma surgeons to diagnose and potentially treat EHDIs via stenting and/or papillotomy, even in the face of previous abdominal surgical procedures [4, 10, 11].

A comprehensive review of 53 cases of EHDIs reported in the English-language literature was conducted, focusing on the evolving role or ERCP in diagnosis and treatment of these injuries. We also report an illustrative case of blunt traumatic injury involving the extrahepatic portion of the left hepatic duct (LHD) and the confluence of the LHD and the right hepatic duct (RHD). Diagnostic and treatment algorithms that incorporate ERCP are presented in order to help systematize and simplify the management of EHDIs.

## 2. ILLUSTRATIVE CASE REPORT

A 26-year old motorcycle rider was struck on his right side by a mid-sized passenger car traveling at approximately 30 miles per hour. He was hemodynamically unstable upon arrival to the hospital (systolic blood pressure 60 mmHg, heart rate 120/min). Bedside abdominal sonogram showed free peritoneal fluid. The patient remained hypotensive despite aggressive fluid resuscitation and was promptly taken to the operating room. He was found to have a large stellate laceration of the liver involving medial segments of the right lobe. Liver was packed and hemostasis was obtained. Splenectomy was performed secondary to splenic laceration that extended into the hilum. A Jackson-Pratt drain was left in the left upper quadrant (LUQ). After a damage control dressing was placed, the patient was taken to interventional radiology where several branches of the right hepatic artery were embolized.

**Figure 1:**
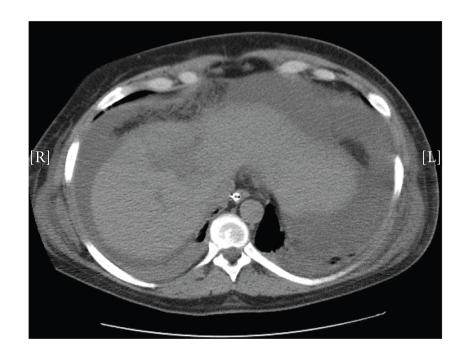
Computed tomographic (CT) scan showing a high-grade liver injury along with large amount of intraperitoneal fluid in the upper abdomen.

**Figure 2:**
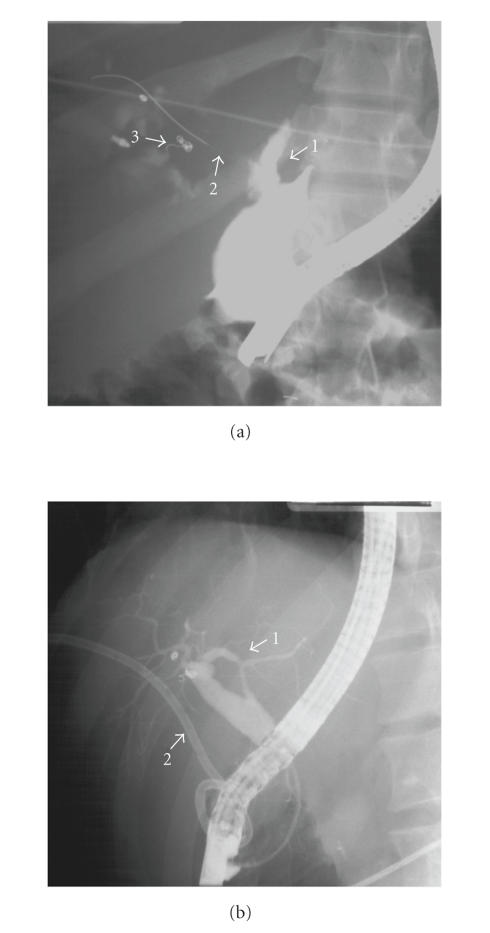
(a) Initial ERCP study demonstrating (1) left hepatic duct transection; (2) wire across the patent right hepatic duct; and (3) embolization coils. (b) Repeat ERCP study demonstrating (1) intact left hepatic duct; and (2) percutaneous drain.

The patient's early hospital course was uneventful and his abdomen was definitively closed on postoperative day two. However, he subsequently began draining increasing amounts of bile from his abdominal drain, associated with concurrent rise in serum bilirubin. Computed tomography (CT) of the abdomen demonstrated a large fluid collection in the upper abdomen (Figure 1). A percutaneous drain was placed into this collection and drained approximately 500 ml of bile. Due to continued drainage of several hundred milliliters of bile per day, an ERCP was obtained. This demonstrated a proximal transection of the extrahepatic portion of the LHD as well as a leak at the confluence of LHD and RHD (Figure 2(a)). Stenting across the transected LHD was attempted but the guidewire could not be passed across the injury. A sphincterotomy was performed and the common bile duct (CBD) was stented in order to decompress the biliary tree.

Over the next several days, the drainage markedly decreased and the patient was discharged to home with drains in place. A repeat ERCP four months after patient's initial injury showed filling of both the RHD and the LHD (Figure 2(b)). His liver function tests (LFTs) at the time were within normal limits. Both the stent and drain were removed, with no subsequent problems reported. He is now four years out from his original trauma, has normal LFTs, and a recent abdominal sonogram showed normal CBD size.

## 3. DISCUSSION

The first case of bile duct rupture due to blunt abdominal trauma was reported by Wainwright in 1799 [13]. Traumatic extrahepatic biliary tree injuries are rare and usually associated with penetrating mechanism [1]. The frequencies of injuries to the biliary tree, in decreasing order, are those of gallbladder, common bile duct (CBD), hepatic ducts (HD) and junction of left hepatic duct (LHD), and right hepatic duct (RHD) [1, 14]. Only 2% of patients with extrahepatic biliary injury have HD injury [1]. We will first discuss the demographics, anatomy, and pathophysiology of EHDIs, followed by a description of some traditional treatment methods and a detailed discussion of the emerging role of ERCP in the treatment of EHDIs, along with diagnostic and treatment algorithms that incorporate ERCP.

**Figure 3:**
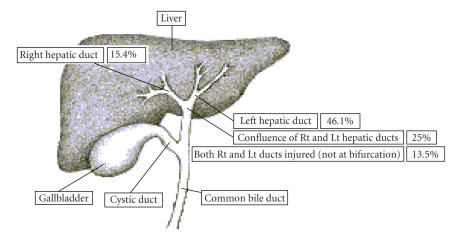
Diagram demonstrating the locations and frequencies of extrahepatic hepatic ductal injuries. Source: [12].

Extrahepatic hepatic ductal injuries (EHDIs) occur predominantly in men, and the male-to-female ratio increases with patient age [2, 12, 15]. Approximately 50% of EHDIs are automobile-related, with the remaining half due to crush injuries, motorcycle crashes, sports/recreational injuries, and falls [12]. The rarity of EHDIs combined with over 50% frequency of severe associated injuries contributes to an average diagnostic delay of about 2 weeks [12, 16]. The frequencies of EHDIs locations are shown in Figure 3.

In EHDIs, the relative fixation of the proximal hepatic ducts to the liver can lead to a shearing force, inducing intraductal hypertension and tearing, as seen with high-speed deceleration [14, 17]. Another mechanism involves compression of the biliary system and gallbladder against the vertebral column and ductal blowout, which may be seen when the gallbladder rapidly empties into a short cystic duct [2, 8, 16]. Ischemic necrosis of the ducts has also been proposed, perhaps accounting for delayed injuries [18]. Extensive ductal dissection during surgery can also produce an ischemic injury. A combination of mechanisms is likely involved in each individual case.

Abdominal ultrasound (AUS) and computed tomography (CT) constitute the initial diagnostic workup. Ductal dilatation and/or periportal fluid collections raise the suspicion of bile duct injury [8]. Percutaneous evacuation of bile can help confirm the diagnosis [9]. In cases of persistent bile drainage, scintigraphy may be useful, although it is poor in pinpointing the site of injury [6, 19]. Cholangiogram is the gold standard for defining a ductal injury [20]. More recently, ERCP has emerged as a valuable adjunct in treatment of EHDI, and can be both diagnostic and therapeutic [12, 21, 22, 23].

One third of EHDIs are missed at initial laparotomy or investigation, and another 2% are not recognized on repeat surgery [8, 12]. Over 50% of patients with EHDIs who do not undergo immediate trauma laparotomy typically have a diagnostic delay of more than 1 day, which can result in significant morbidity and mortality [2, 8]. Sterile bile causes minimal peritoneal reaction, with vague abdominal pain and distention, nausea, vomiting, and jaundice [8, 24]. Liver injury is the most common associated injury (55% of patients), followed by extremity (19%), pelvic fractures (17%), and splenic and gallbladder injury [12]. Pneumothorax, rib fractures, and head injury are less frequent. Others report combined injuries involving the duodenum, stomach, colon, pancreas, and non-EHDI biliary duct injuries (5%) [12, 25].

Mortality was reported in 3.8% to 12.7% cases of EHDI, with blunt injuries being associated with higher mortality than penetrating injuries [1, 2, 12, 25, 26]. EHDIs are associated with long hospitalizations (average of 40 days) and many potential complications [12]. Early complications include wound infection, abscess, and biliary fistula [8, 27, 28]. Late complications include ductal stricture with or without cholangitis and posttraumatic hepatic atrophy [25, 29, 30].

When the diagnosis of EHDIs occurs during an emergent celiotomy, the primary focus should be on patient stabilization, hemostasis, temporizing stenting, ligation, and T-tube placement should be considered [1, 8, 31]. At the very least, the injured duct should be tagged and the area drained, with definitive repair performed later. Long-term stenting across injured hepatic ducts may be considered, even without suture repair [17, 32]. Choledochoenterostomy and hepaticoenterostomy have been used for major ductal injuries, including complete transections [1, 15, 33]. When performing biliary reconstruction, the size of the duct and viability of its blood supply have to be considered, and end-to-end ductal repair should be avoided in complete injury due to the risk of stricture formation [8, 16, 25]. Other techniques include repair with vein, serosal or jejunal patch [33,34]. Adequate operative drainage is essential [3]. Bilioenteric anastomoses produce good long-term outcomes in 85–90% of cases [35–38]. Long-term anastomotic stenting (6–9 months) has been supported by some authors [25, 36], while others stent for a shorter period of time or not at all [35, 39]. Anastomotic stents decompress the biliary tree, allow postoperative radiographic followup, and there may be a correlation between outcome and the time stented as anastomotic catheters may limit the contraction of collagen and stricturing [25]. Opponents of stenting argue that stents contribute to complications (stent dislodgment, occlusion by biliary debris, and cholangitis) [25, 40]. While many biliary strictures appear in the first 2 postoperative years, it may take up to 5 years for 80% of strictures to occur, with approximately 20% of failures after that period, suggesting that a long-term followup of 7–10 years may be optimal [25, 35, 41]. Tacking of the Roux-en-Y jejunal loop marked with metallic clips to the abdominal wall can help facilitate future biliary tree access [36].

## 4. EMERGING ROLE OF ERCP IN MANAGEMENT OF EHDI

Management of EHDIs depends on the patient's overall clinical status, associated injuries, and the location and extent of the injury [8]. Patients who are hemodynamically stable on initial presentation and do not require immediate surgical intervention can safely undergo nonoperative management of bile duct injuries—an attractive therapeutic alternative [42, 43]. This is further supported by the use of ERCP in treatment of iatrogenic extrahepatic bile duct injuries, which is well described and accepted [12, 22].

More recently, ERCP has emerged as a valuable adjunct in treatment of EHDI, and has been used to define the anatomy of the injury as well as to provide definitive therapy [12, 21–23, 43]. In fact, a total of 19 cases in this review involved ERCP utilization in either diagnostic or therapeutic capacity (Table 1). The safety and efficacy of ERCP has been advocated in increasing number of publications, with excellent (>90%) ductal visualization success and low (<10%) morbidity [7, 43].

Endoscopic retrograde cholangiopancreatography has been successfully utilized in treatment of hepatic ductal injuries both as a primary treatment modality and as an adjunct to surgery, with some of the patients having previously undergone at least one laparotomy [43]. Indeed, it may be that ERCP is the optimal choice for treatment of bile duct injuries regardless of whether the patient underwent recent surgery. One might speculate that performing a potentially therapeutic ERCP for EHDIs in the setting of a recent laparotomy may actually constitute the safest initial approach, given the possibility of postoperative adhesions and the risk of bile duct devascularization due to surgical dissection. In fact, at least one reported death was due to massive hepatic bleeding encountered during an operative attempt at repair of RHD stricture [25]. The usefulness of ERCP in such setting is exemplified by the current case, where the diagnosis of EHDI was not made until after the initial trauma laparotomy, and reoperation to restore biliary continuity would have been very difficult and risky. Not only did ERCP confirm the diagnosis of EHDIs and facilitated definitive treatment of the injury, but also indirectly pointed to the potential cause of surgical failure if operative management was attempted—small transected LHD that could not be traversed with the guidewire. Because surgical repair of small extrahepatic bile ducts can be exceedingly difficult [62, 63], a topic beyond the scope of this discussion, ERCP may be the preferred treatment method in this scenario as well.

In majority of reported cases, including the current report, ERCP-facilitated ductal stenting was performed [8, 10, 16, 43]. In fact, ERCP with sphincterotomy and drainage avoids surgery in 70–90% of iatrogenic ductal injuries by reducing the biliary intraductal pressure gradient [42]. Percutaneous drainage of any bile collections should be performed as well, with prophylactic drainage suggested by some even in the absence of an active bile leak [43]. In the current case, while a CT scan revealed a fluid collection, it failed to fully delineate the anatomic injury. Much like in other reports of both traumatic and nontraumatic bile duct injuries, ERCP was used to define the anatomy of the injury and to treat it definitively with biliary decompression and stenting [12, 21–23]. It is likely that endoscopic stenting provides similar effects to operative anastomotic stenting via biliary decompression and by potentially decreasing stricturing through limiting collagen contraction [25]. Failures of endoscopic therapy are rare, and have been associated with leaks from noncommunicating or anatomically “excluded” ductal injuries [42].

A recent review of EHDIs with an average followup period of 26 months reported an increasing use of ERCP in both diagnostic and therapeutic capacities [12]. Among patients treated primarily with ERCP, 9/19 had followup studies [4, 8, 11, 12, 16, 43, 61]. Among these patients, 8/9 showed resolution of biliary leak and no evidence of biliary stricture [4, 8, 11, 16, 43, 61] and 1/9 showed nonvisualization of the previously injured LHD [11]. As more long-term followup data confirm good clinical results, the ERCP will likely take the dominant position as the initial treatment of choice for EHDIs.

Complication rates associated with ERCP use in the setting of bile duct injuries are low (<10%) [7]. Reported post-ERCP complications include pancreatitis and persistent hyperamylasemia [10, 14]. In addition, stent migration or clogging may occur [43]. In adult patients, stent migration has been noted in upto 5% of patients [10]. Stent clogging is more common, with upto 30% incidence within 3 months of stent placement [10]. There is also a low risk of infection and bleeding related to percutaneous catheter drainage of EHDI-associated bile collections [43]. Ductal stenosis at the site of injury is an important late complication of ERCP and stenting. It has been postulated that prolonged stenting (up to 12 months), sometimes with multiple stents, may provide both treatment and prevent further stricturing [42].

**Figure 4:**
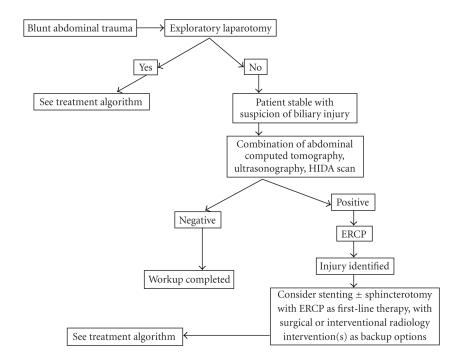
Proposed diagnostic algorithm for extrahepatic hepatic ductal injuries. ERCP = endoscopic retrograde cholangiopancreatography. HIDA = nuclear biliary scan.

**Figure 5:**
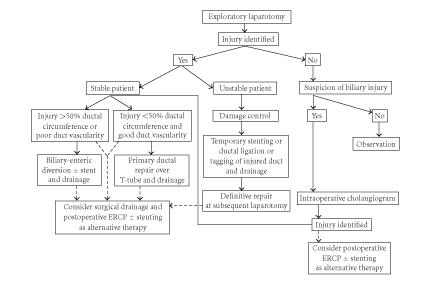
Proposed treatment algorithm for extrahepatic hepatic ductal injuries.

After a literature search was conducted, Table 1 was constructed to summarize all known cases of EHDIs from 1952 to 2006. Based on our case as well as the literature search, proposed diagnostic (Figure 4) and treatment (Figure 5) algorithms were designed in order to systematize clinical decision-making in the setting of EHDIs. These algorithms reflect the evolving role of ERCP in treatment EHDIs.

## 5. CONCLUSIONS

Because the clinical presentation is often insidious, EHDIs are frequently missed on the initial clinical evaluation. The management of EHDIs has changed over the last decade. Availability of ERCP presented trauma surgeons with a new diagnostic and therapeutic alternative. With good short-term results of ERCP being well established, a growing body of data is demonstrating equally good results on long-term followup. We recommend the use of diagnostic and treatment algorithms to standardize care, decrease diagnostic delay, and potentially improve outcomes.

## Figures and Tables

**Table 1:** Collected summary of all reported cases of extrahepatic hepatic ductal injuries from 1925 to present.

Date, Author, (Ref.) (chronological)	Age (y.)	Gender	Mechanism of injury	Nature of ductal injury	Treatment	ERCP
1925, Cope [44]	10	M	MVC	Confluence of R & LHD	Cholecystostomy drains	N

1929, Long [45]	40	M	Crushed between autos	Confluence of R & LHD	Cholecystostomy drains	N

1938, Lewis [27]	49	M	MV versus PED	Confluence of R & LHD	Drainage. Followed by re-drainage	N

1953, Walker [46]	2	M	Run over by a tractor	Confluence of R & LHD	R-en-Y repair over stents	N

1955, Baty [47]	25	M	MVC	LHD laceration	Common duct T-tube	N

1955, Schaer [48]	50	M	Struck by a bull	RHD lacerated anteriorly (0.5 cm)	CBD stent, drains	N

1956, Foman [49]	34	M	MVC	R & LHD near the confluence	Cholecystostomy, drain	N

1961, Nikishin [50]	3	M	Run over by an auto	RHD laceration	Drains	N

1964, Hartman [51]	2	F	MV versus PED	Confluence of R & LHD	Cholecystostomy with drainage	N

	6	M	MVC	(1) Bile duct leak at unknown site (2) LHD transection	Drains, primary repair over catheters, common duct tube, cholecystostomy, feeding jejunostomy	N

1967, Noone [52]	8	M	Bicyclist falling onto handle bars	(1) Lacerated R liver lobe (2) R & LHD disruption	Primary anastomosis over catheters, cholecystostomy, drains	N

1967, Sewell [53]	14	F	MVC	LHD avulsion	LHD ligation, T tube	N

1968, Maier [26]	37	M	MCC	RHD laceration (lateral)	Repair over T tube	N

1969, Haynes [54]	N/A	N/A	Blunt abdominal trauma	(1) R hepatic lobe laceration (2) LHD laceration	Drains, Primary ductal repair	N

1969, Estrada [55]	26	M	MVC	LHD laceration, posterior	Repair over T-tube	N

1972, Zollinger [32]	21	F	MVC	R & LHD laceration	Repair over catheters, drain	N

	48	M	MVC	LHD avulsion	Drains, RHD anastomoses to R-en-Y, stent, T-tube	N

1974, Williams [56]	3	M	MV versus PED	LHD avulsion	End-to end anastomosis	N

1980, McFadden [28]	31	M	MVC	Combined R & LHD	Hepaticojejunostomy	N

1985, Jones [21]	37	M	MCC	Confluence of R & LHD	R & L hepaticojejunostomy	N

1985, Michelassi [2]	9	M	Patient denied any trauma	LHD partially severed	Suture repair. Drains. T-tube	N

1987, Salam [19]	17	F	MVC	RHD laceration	Suture repair	N

1991, Dawson [3]	17	M	Crushed by a log	LHD avulsion, 3 cm tear across the junction of CHD and RHD	Suture repair of RHD & CHD, R-en-Y hepaticojejunostomy	N

1991, Monk [34]	14	M	Bicycle crash	LHD disruption (noncircumferential)	Vein patch cholangioplasty with stent & drainage	N

1992, Muin [24]	45	M	Hit by falling tree branch	Confluence of R & LHD (superiorly)	R-en-Y hepaticojejunostomy	N

1993, Hills [30]	18	F	MVC	LHD injury	Percutaneous stent	N

	15	F	MVC	LHD injury	Cholecystectomy, omental plug	N

	16	M	MCC	LHD injury	Partial liver resection	N

1993, Moulton [10]	5	F	MV versus PED	LHD tear	Stent placed via ERCP	Y

1994, Brenneman [57]	36	M	MCC	LHD injury	Repair over T-tube	N

1995, Gerndt [8]	20	M	MVC	L & RHD injury	Primary repair. Drains.	Y

	19	M	MVC	LHD transection	Drains, R-en-Y hepaticojejunostomy	N

	21	M	MVC	(1) L & RHD injury near bifurcation (2) Transected lateral LHD	ERCP with stenting of R ductal system	Y

1995, Baer et al. [58]	31	M	Fell 10 meters	LHD injury	Drains	N

1995, Poli [9]	12	F	Kicked by a horse	Confluence of R & LHD, CHD tear	Nasobiliary and percutaneous drains	Y

1996, Eid [4]	21	M	Crushed by a container	ERCP, LHD tear	Stenting via ERCP	Y

1996, Hayakawa et al. [59]	21	M	MCC	LHD transection	Primary repair over stent	N

1996, Sharma [5]	35	M	Fall from a height	RHD bile leak	Endoscopic papillotomy	Y

1997, Sakamoto [17]	23	M	Fall from ladder	Confluence of R & LHD	Drains at laparotomy	N

	22	M	MV versus PED	LHD laceration	Stent at laparotomy	N

1999, Arkovitz [6]	7	M	MV versus PED	(1) Complete avulsion of LHD (2) Attenuated RHD	Stenting, Drainage, L and R hepaticojejunostomies	Y

1999, Simstein [60]	21	M	Pinned under automobile	(1) Injury at R & LHD confluence (2) RHD disruption	Intraoperative placement of drains	Y

1999, Bin Yahib et al. [14]	3	M	MV versus PED	Torn R & LHD	Primary repair of R & LHD, R-en-Y hepaticojejunostomy	Y

2000, Sanders [7]	11	M	All terrain vehicle accident	LHD injury	Cholecystostomy tube, Jackson-Pratt™ drains	Y

2001, D'Amours [61]	34	M	Fall 9 meters	R & LHD injury	ERCP. Sphincterotomy and stenting	Y

	41	M	MVC	LHD injury	Drains, ERCP with sphincterotomy and double pigtail stent	Y

2001, Nuzzo [29]	42	F	MVC	(1) LHD transection (2) LHD stricture	LHD end-to-end anastomosis. ERCP stenting and serial dilations of LHD stricture	Y

2001, Rodriguez-Montes [25]	N/A	N/A	N/A	RHD laceration	T-tube, RHD R-en-Y choledochojejunostomy	N

	N/A	M	N/A	(1) LHD transection (2) RHD stricture (delayed finding)	Primary repair of LHD transection. Endoscopic stenting od RHD.	Y

2002, Sharpe [11]	11	M	Sledding accident	Transected LHD	Percutaneous drainage of subhepatic space and transampullary stent	Y

2003, Nathan [16]	17	M	MVC	Confluence of R & LHD	Intraoperative placement of drains. ERCP with stent placement	Y

2006, Almaramhi [43]	6	F	MVC	RHD	ERCP with stent placement and percutaneous drainage	Y

	6	M	MVC	RHD	ERCP with stent placement and percutaneous drainage	Y

Current case	26	M	MCC	(1) Confluence of R & LHD (2) LHD injury	External drainage, ERCP with sphincterotomy and CBD stenting	Y

Abbreviations: N/A = Data Not Available; ERCP = Endoscopic retrograde cholangiopancreatography; CHD = Common hepatic duct; LHD = Left hepatic Duct; RHD = Right hepatic duct; R & LHD = Right and Left Hepatic Ducts; R = Right; L = Left; R-en-Y = Roux-en-Y; MVC = Motor vehicle crash; MV versus PED = Motor vehicle versus pedestrian.
